# Treatment effect and safety of seltorexant as monotherapy for patients with major depressive disorder: a randomized, placebo-controlled clinical trial

**DOI:** 10.1038/s41380-024-02846-5

**Published:** 2024-12-11

**Authors:** Sofie Mesens, Iva Kezic, Peter Van Der Ark, Mila Etropolski, Gahan Pandina, Heike Benes, Adam Savitz, Wayne C. Drevets

**Affiliations:** 1 Janssen Research & Development, Beerse, Belgium; 2 Janssen Research & Development, Titusville, NJ USA; 3 Somni Bene, Institut für Medizinische Forschung und Schlafmedizin Schwerin GmbH, Schwerin, Germany; 4 Janssen Research & Development, San Diego, CA USA; 5 Present Address: Currently at GSK Consumer Healthcare, Collegeville, PA USA; 6 Present Address: Currently at Weill Cornell Medical College, White Plains, NY USA

**Keywords:** Depression, Diagnostic markers, Diseases

## Abstract

The antidepressant efficacy and safety of seltorexant monotherapy in major depressive disorder (MDD) was investigated in a placebo-controlled, placebo lead-in, randomized, double-blind, phase 1b study. Participants were randomized to receive seltorexant (20 mg or 40 mg) or placebo. The treatment effect was assessed by changes in the Hamilton Rating Scale for Depression-17 item (HDRS_17_) from treatment-period baseline to week 5 in lead-in placebo non-responders (“enriched” intent-to-treat analysis set). As a secondary outcome, the effect of seltorexant on HDRS_17_ was assessed in patients with and without subjective insomnia. Seltorexant’s effects on polysomnography, serum cortisol, and cortisol waking response were also measured. In total, 128 participants were enrolled, including 86 in the enriched sample (lead-in placebo non-responders). The mean changes from baseline (SD) in HDRS_17_ score at week 5 differed significantly across arms: −7.0 (5.04) for seltorexant 20 mg, −5.5 (4.34) for seltorexant 40 mg, and −4.4 (3.67) for placebo (p = 0.0456), which was attributable to the difference between the 20 mg and placebo arms (p = 0.0049). Improvement in depression severity at week 5 for seltorexant 20 mg was greater in patients with higher baseline insomnia severity (nominal p = 0.0059). The treatment benefit in the 20 mg arm remained significant when HDRS scores were adjusted by removing the sleep items (nominal p = 0.0289). The mean HDRS_17_ change versus placebo was numerically larger in the 20 mg than the 40 mg arm, consistent with data from a previous study in which seltorexant was administered adjunctively to conventional antidepressants. In secondary analyses, the waking cortisol response decreased in the 20 mg arm but not the 40 mg or placebo arms, and while total sleep increased more in the 40 mg arm, this arm also showed reduced REM onset latency and increased stage N1 sleep, which were not evident in the 20 mg arm. These biomarker data suggest mechanistic hypotheses that may account for the apparent curvilinear dose-response relationship of seltorexant. Trial Registration: ClinicalTrials.gov, NCT03374475.

## Introduction

Seltorexant (JNJ-42847922) is a selective orexin-2 receptor (OX2R) antagonist being developed as a treatment for major depressive disorder (MDD). Orexins are excitatory neuropeptide transmitters produced in orexinergic neurons in the lateral hypothalamus, which project to neural circuits that regulate sleep-wake cycles, monoamine neurotransmitter release, autonomic function, hypothalamic-pituitary-adrenal (HPA) axis activation, and emotional behavior [1, 2]. Orexins mediate behavior, glucocorticoid hormone release, and sympathetic autonomic activation under conditions of high motivational relevance, such as stress, threat, and opportunities for reward [1, 3]. For example, orexins activate monoaminergic neurons projecting to the lateral hypothalamus, basal forebrain, and cerebral cortex to exert arousal effects, and during wakefulness the tonic excitation of orexin neurons by limbic input may be enhanced by stress or emotional stimulation [1, 4, 5]. Some aspects of these roles are differentially mediated by orexin 1 vs orexin 2 receptor signaling [2]. For example, in mice both genetic and pharmacological inhibition of OX2R (but not of OX1R) signaling blocked the stress-induced increment in corticotropin release [3, 6].

A major subgroup of individuals with MDD manifests hyperarousal which contributes to difficulties falling and staying asleep as well as to HPA-axis overactivation [7–10]. While sleep disturbance associated with depression can occur in either direction (i.e., insomnia or hypersomnia), the majority of MDD patients suffer from insomnia, and manifest characteristic polysomnographic changes including impaired sleep continuity, disinhibition of REM sleep, shortened REM latency and changes in non-REM sleep [11]. Moreover, chronic or recurrent insomnia commonly precedes the development of major depressive episodes as a potential risk factor or clinical antecedent [12].

In clinical studies the administration of seltorexant produced antidepressant and sleep promoting effects in MDD. In an initial exploratory study in antidepressant-treated MDD patients with persistent insomnia, a single dose of seltorexant significantly decreased the latency to persistent sleep, and increased total sleep time (TST) and sleep efficiency (SE) [13]. In a second study in MDD patients, administration of seltorexant 20 mg at bedtime for 10 days was associated with a significant improvement of core depressive symptoms (assessed using the Hamilton Rating Scale for Depression-17 [HDRS_17_] score adjusted by removing sleep items and by the HDRS-6) compared to both placebo and diphenhydramine [nocebo] administration [14, 15]. In that study, depressive rumination ratings (assessed by the Ruminative Response Scale (RRS) were significantly associated with both depression severity and an increase in the duration of stage 1 sleep (“light sleep”) at baseline, More recently, in a 6-week double-blind, placebo-controlled study, 20- but not 10- or 40-mg seltorexant at bed time showed significant efficacy as an adjunctive treatment to an SSRI/SNRI in MDD patients who had experienced inadequate clinical response to the antidepressant [6]. The improvement in depression severity at week 6 for seltorexant 20 mg was greater in patients with moderate-severe insomnia at baseline, assessed using the Insomnia Severity Index (ISI).

The current phase 1b study characterized the antidepressant efficacy of seltorexant administered as a monotherapy in depressed participants with MDD. The primary objective was to determine the magnitude of treatment effect (seltorexant vs. placebo) on symptoms of depression measured by HDRS_17_ in lead-in placebo non-responders. A secondary objective was to investigate differential effects of seltorexant as compared to placebo on HDRS_17_ in patients with and without subjective insomnia. Moreover, the mechanisms that may underlie seltorexant’s antidepressant effects and dose response relationship were explored by evaluating its effects in depressed participants with high versus low levels of rumination, and on polysomnography and serum cortisol levels.

The doses tested in the current trial were selected based on the results of previous trials using seltorexant in MDD. In the initial exploratory study in antidepressant-treated MDD patients with persistent insomnia cited above, single doses of seltorexant at 10-, 20-, and 40-mg significantly decreased the latency to persistent sleep, and increased TST and SE, with the 20- and 40-mg doses providing comparable efficacy on these sleep measures [13]. In the 6-week double-blind, placebo-controlled study, 20- but not 10- or 40-mg seltorexant qhs showed significant efficacy when administered adjunctively to an SSRI/SNRI in MDD patients, although the 40 mg dose showed similar antidepressant response and remission rates as the 20 mg dose [6]. The current study was designed to assess seltorexant’s antidepressant efficacy as a monotherapy, and to assess whether the trend toward greater efficacy observed at the 20 mg dose as compared to the 40 mg dose was reproducible.

## Methods

### Trial design

**Fig. 1:**
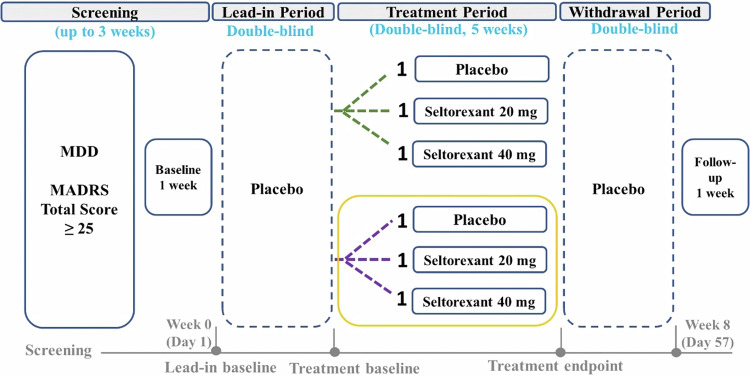
Study Design. This phase 1 study consisted of a screening phase (up to 3 weeks), a double-blind treatment phase (8 weeks), and a follow-up phase (1 week). The double-blind treatment phase consisted of 3 periods: a blinded placebo lead-in period, a treatment period (5 weeks), and a blinded placebo withdrawal period. The study duration for each patient was ~13 weeks. MDD, major depressive disorder; MADRS, Montgomery–Åsberg Depression Rating Scale; HDRS_17_, Hamilton Depression Rating Scale-17; green dotted line, placebo responders based on reduction from lead-in baseline in HDRS_17_; purple dotted line, placebo non-responders based on reduction from lead-in HDRS_17_; gold solid line, contributes to primary analysis.

This multicenter, placebo-controlled, randomized, double-blind, study assessed the efficacy, mechanism of action, and safety of seltorexant in MDD patients not currently receiving antidepressant therapy. Briefly, the study consisted of a screening phase (up to 3 weeks), a double-blind treatment phase (8 weeks), and a follow-up phase (1 week) (Fig. 1). The double-blind treatment phase consisted of 3 periods: a blinded placebo lead-in period, a treatment period (5 weeks), and a blinded placebo withdrawal period. The study duration for each patient was ~13 weeks. Fourteen centers across 5 countries (Belgium, Germany, United Kingdom, Netherlands, and the US) were involved. The study protocol and amendment(s) were reviewed by an Institutional Review Board.

### Randomization and blinding

Patients were randomized 1:1:1 to receive placebo, 20-, or 40-mg seltorexant. Randomization occurred after the placebo lead-in period and was balanced using randomly permuted blocks stratified by lead-in placebo response status and presence of moderate-severe subjective insomnia symptom severity (defined as ISI score >15) and subjective sleep disorder (as documented in the sleep diary by subjective Sleep Onset Latency (SOL) > 30 min and TST of <6 h at least 3 nights over 7 recorded days). Investigators and patients remained blind to the duration of the lead-in and withdrawal periods (combined 21 days) and to the lead-in placebo response criteria.

### Dosage and administration

Visually identical capsules were self-administered (2 capsules orally, once daily) over 8 weeks, 15 min before bedtime, and consisted of 20 mg seltorexant (2 capsules of 10 mg), 40 mg seltorexant (2 capsules of 20 mg), or placebo (2 capsules).

### Study population

Participants with MDD per Diagnostic and Statistical Manual of Mental Disorders, Fifth Edition, without psychotic features, and moderate to severe depression severity assessed by a Montgomery-Åsberg Depression Rating Scale total score ≥25 at screening were enrolled. Participants had not received antidepressant pharmacotherapy for at least 2 weeks before screening. Participants were not withdrawn from current antidepressant treatment for the purpose of the study. Volunteers were excluded from participation if they had experienced clinically significant suicidal behavior or serious suicidal ideation within 6 months, moderate or severe substance abuse disorder, primary or current diagnosis of other psychiatric conditions (ie. primary diagnosis of anxiety disorders, bipolar disorder, psychotic disorder), or lack of response to more than 2 treatments in the current episode (<25% response; Additionally, women of childbearing potential (WOCBP) were excluded. Treatment with sedative hypnotics of the benzodiazepine or GABA_A_ receptor agonist classes, opioid analgesics or antipsychotic agents was not allowed from screening throughout the study. Ongoing psychotherapy (if initiated more than 2 months prior to randomization) was allowed. The study protocols were reviewed by an independent ethics committee or institutional review board at each site (e.g., EC UZA [reference number: 17/43/491]; CCMO NL [reference number: NL63487.056.17]) and conducted following the principles of Good Clinical Practice derived from the Declaration of Helsinki, and in accordance with local regulations and International Council of Harmonization guidelines. Patients or their legal representatives provided their written informed consent to participate in the study.

### Analysis sets

The “enriched” intent-to-treat analysis set (eITT) evaluated for efficacy was defined as all enrolled lead-in placebo non-responders (placebo response was defined by ≥30% reduction from baseline in HDRS17 total score at the end of the placebo lead-in period) randomized into the treatment period who received ≥1 dose of study medication and had ≥1 post-baseline HDRS_17_ assessment during the treatment period. Safety information was summarized using the full safety analysis set, defined as participants who received ≥1 dose of study medication.

### Sample size determination

An estimated 26 patients in each treatment group (78 total) were planned to complete the study in the enriched patient population after excluding placebo-responders in the placebo lead-in phase. The study sample size chosen (N = 78 non-placebo responders completing the study) was expected to allow investigation of the potential effect of seltorexant relative to placebo on symptoms of depression based upon power calculations that used data from a previous study conducted using the same compound in a similar patient population [15]. Assuming a standard deviation (SD) of 5.8 for the treatment difference in HDRS_17_ total score observed in this previous study [15], the calculated sample size allowed detection of a difference between any seltorexant group and placebo of 3.4 points and 4.2 points, respectively, at 20 and 10% one-sided significance levels with 90% power. This translated to effect sizes of 0.59 and 0.72, respectively, which were comparable to the effect size previously observed [15].

To achieve this eITT sample size, 138 participants were planned to enter the lead-in period. After adjusting for an estimated placebo response rate of 25%, drop-out rate during the lead-in period of 10%, and drop-out rate during the treatment period of 15%, a total of 93 placebo non-responders were planned for randomization to receive either placebo, 20 mg or 40 mg seltorexant in 1:1:1 ratio.

### Efficacy variables

The key efficacy variable for the primary analysis was the mean change in HDRS_17_ total score from treatment baseline through treatment Week 5. The efficacy variables for the key secondary analyses were the change in HDRS_17_ score from treatment baseline through treatment Week 5 by baseline ISI score (>15 defining moderate to severe insomnia symptoms) and presence of subjective sleep disorder (presence, absence) defined as ISI score >15 *plus* sleep diary documentation of subjective SOL > 30 min and TST < 6 h (during at least 3 nights over 7 recorded days). Exploratory efficacy endpoints were: change in HDRS_17_ score from treatment baseline to treatment Week 5 by severity of ruminations (rated by baseline Rumination Response Scale [RRS]) score <50 or ≥50 defining higher rumination level based on analysis results in phase 2 MDD study) [16–18]; remission (HDRS_17_ score ≤ 7) and response rates (≥50% improvement in HDRS_17_ total score from treatment baseline) over the treatment period; change in “core” depressive symptoms using the 6-item subscale from HDRS_17_ (HAM-D_6_; which is limited to the items assessing depressed mood, guilt, work and activities, psychomotor retardation, psychic anxiety, and general somatic symptoms) during the treatment period [15]; change in HDRS_17_ score “adjusted” by removing the three sleep related items; changes in Quick Inventory of Depressive Symptoms-16 (QIDS-SR16) score (1-week recall period) from treatment baseline to treatment Week 5 [19]. Safety assessments are outlined below.

Other efficacy variables included: (1) levels of cognitive arousal/distress (mean changes from baseline in RRS), (2) cortisol measures (overnight sampling of serum cortisol level during the lead-in baseline phase and at Week 3/4), (3) Sleep assessments (polysomnography [PSG] assessments during overnight stay in the study center at the lead-in baseline and at week 3/4.

### Cortisol assessments

Cortisol waking response was defined as a change from morning baseline cortisol level (‘Light On’ measurement) to ‘Light On+30 min’. Nadir cortisol levels were defined as the average cortisol level from “light out” to 4 h after “light out” to emphasize the period of seltorexant’s most substantial pharmacodynamic activity (t_1/2_ = 2–3 h) [20, 21].

### PSG assessments

Patients were instructed to go to bed between 10:00 pm and 12:00 am (midnight) and remain in bed for 8 h. PSG equipment was used to record sleep EEG along with electromyography, ECG and electro-oculography (collectively referred to as PSG). PSG scoring was based on the guidelines of the American Academy of Sleep Medicine and was assessed at a blinded laboratory [22].

### Statistical methods

The primary hypothesis tested was that seltorexant is superior to placebo in the treatment of MDD when given as a monotherapy. A secondary objective was that the antidepressant treatment effect would be greater in patients with moderate-severe insomnia. The effect of seltorexant on symptoms of depression during the treatment period was evaluated using change from treatment baseline in HDRS_17_ total score, HDRS_17_ Sleep Item-Adjusted score and HDRSAM-_6_ score during the treatment period. [14].

The primary hypothesis was tested by comparing the changes in HDRS_17_ total score after treatment Week 5 using a mixed-effects model for repeated measures (MMRM), with subject as random factor; presence of subjective sleep disorder, time, treatment (placebo, seltorexant) and time-by-treatment interaction as factors and treatment baseline score as a continuous covariate. The secondary analyses were performed by comparing changes in HDRS_17_ total score after Treatment Week 5 and changes in HAM-D_6_ subscale score at Treatment Week 5 by subgroups using an analysis of covariance (ANCOVA) model with treatment, baseline subgroup value (subjective sleep disorder, ISI, or RRS score) and baseline subgroup value-by-treatment interaction as factors and treatment baseline HDRS_17_ total score as a continuous covariate. No multiplicity adjustment was performed for the secondary analyses. Changes in cortisol waking response (see Supplement for details of the methods) were analyzed using an ANCOVA model with treatment as factor and lead-in baseline cortisol waking response as a continuous covariate. The HDRS_17_ remission and response rates along with changes in RRS score and PSG parameters were analyzed descriptively.

### Safety assessments

Safety evaluations included adverse events, vital signs, ECGs, and laboratory data safety assessments. Suicidal ideation and behavior were assessed by Columbia Suicide Severity Rating Scale (CSSRS) over the treatment period and change at treatment Week 5. A

## Results

### Study population

A total of 128 MDD patients were enrolled. Enrollment was discontinued before the anticipated 138 patients based on the lower-than-expected dropout and placebo responder rates. Of these, 127 patients were included in the safety population, of whom 86 were in the eITT set and 40 were lead-in placebo responders. Patients were recruited between January 9, 2018, and February 6, 2019.

Demographic and clinical characteristics are summarized in Table 1. A total of 50 (58.8%) of the eITT patients were diagnosed with moderate to severe subjective sleep disorder. The mean (SD) baseline ISI score was 17.4 (4.40). The treatment groups were similar with respect to the baseline characteristics. Of the 86 patients in the eITT population, 79 (91.9%) completed the study. The dropout rate was 3.3% in the placebo group and 10.7% in each seltorexant dose group.

**Table 1:** Demographics and patient characteristics: eITT analysis set.

	Placebo	Seltorexant (20 mg)	Seltorexant (40 mg)	Total
Age (Years), N	30	28	28	86
Mean (SD)	39.5 (9.26)	37.9 (10.19)	38.6 (11.63)	38.7 (10.28)
Median	39.0	37.0	38.0	38.0
Range	23; 55	21; 55	20; 55	20; 55
Gender, N	30	28	28	86
Female (%)	6 (20.0)	8 (28.6)	7 (25.0)	21 (24.4)
Male (%)	24 (80.0)	20 (71.4)	21 (75.0)	65 (75.6)
Presence of subjective sleep disorder, N	29	28	28	85
No (%)	11 (37.9)	14 (50.0)	10 (35.7)	35 (41.2)
Yes (%)	18 (62.1)	14 (50.0)	18 (64.3)	50 (58.8)
HDRS_**17**_^a^, N	30	28	28	86
Mean (SD)	17.7 (5.11)	18.1 (4.88)	17.8 (4.18)	17.9 (4.69)
Median	17.0	18.0	17.5	17.5
Range	8; 32	9; 25	12; 27	8; 32
Race, N	30	28	28	86
American Indian or Alaska Native	0	0	0	0
Asian (%)	0	2 (7.1)	1 (3.6)	3 (3.5)
Black or African American (%)	1 (3.3)	0	0	1 (1.2)
White (%)	29 (96.7)	26 (92.9)	27 (96.4)	82 (95.3)

HDRS_17_ Hamilton Rating Scale for Depression-17 item.

^a^Treatment baseline.

### Efficacy

#### Changes in HDRS_17_ total score over treatment period: eITT analysis set

**Fig. 2:**
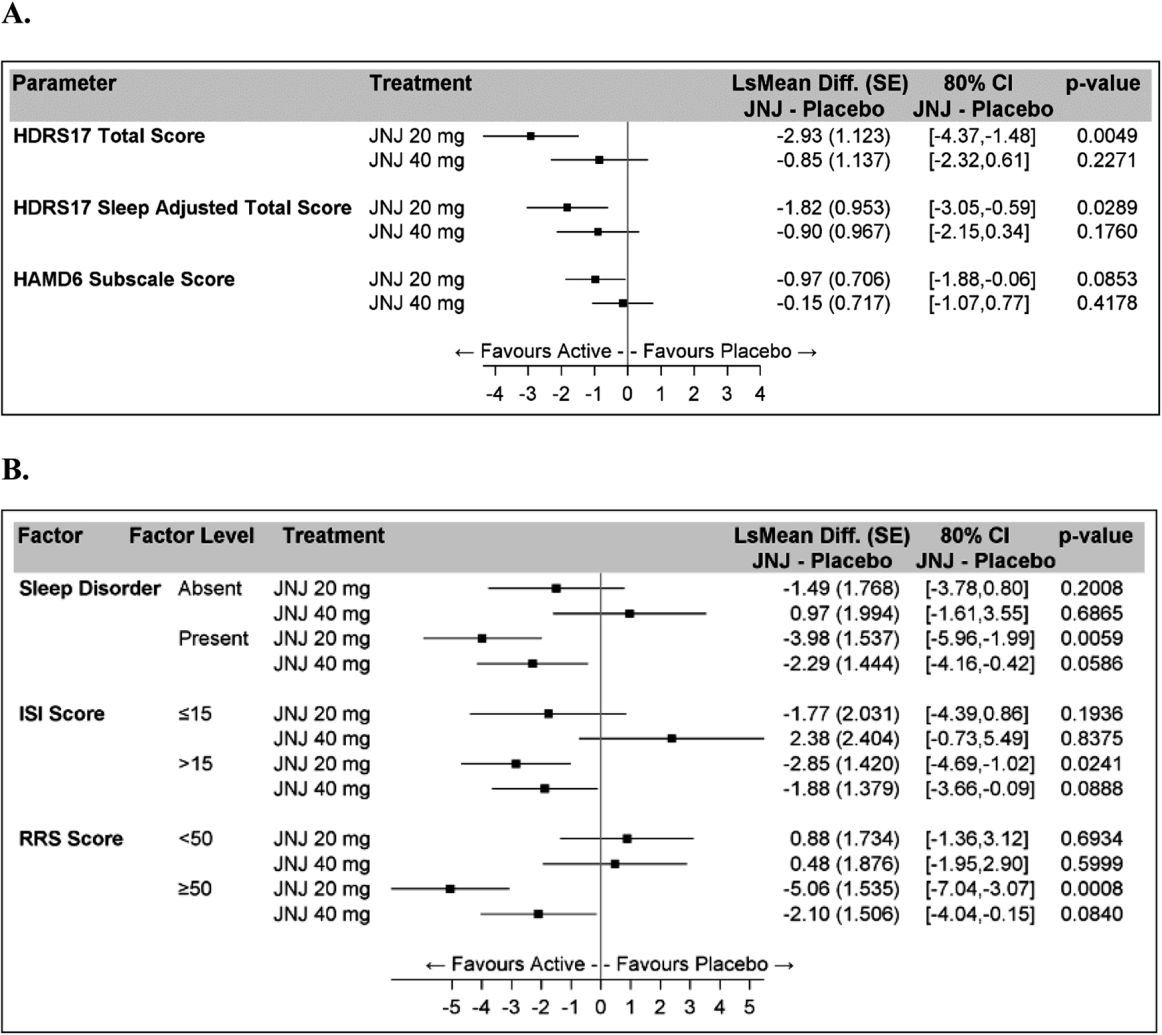
Evaluation of HDRS_17_ in the eITT analysis set. **A** Changes in HDRS_17_ Sleep Adjusted Total Score and HAM-D_6_ Subscale Score; eITT Analysis Set. **B** HDRS_17_ Total Score: Least Squares Mean Changes from Baseline During the Treatment Period; eITT Analysis Set. Sleep disorder as documented in the sleep diary by subjective Sleep Onset Latency >30 min and total sleep time of <6 h at least 3 nights over 7 recorded days. eITT, “enriched” intent-to-treat; HAM-D_6_, 6-item subscale from HDRS_17_; HDRS_17_, Hamilton Rating Scale for Depression-17 item; ISI Insomnia Severity Index, JNJ, seltorexant, RRS Rumination Response Scale, SE standard error.

The mean (SD) HDRS_17_ total score at Treatment Period baseline (i.e., following the blinded placebo Lead-In Period; Fig. 1) was 17.9 (4.69). The mean change from Treatment Period baseline (SD) in HDRS_17_ total score at Treatment Week 5 was −7.0 (5.04) for Seltorexant 20 mg, −5.5 (4.34) for Seltorexant 40 mg and −4.4 (3.67) for placebo. Based on the results from the MMRM model, a significant positive efficacy signal was detected for seltorexant across dose levels versus placebo (*p* = 0.0456). The LSMean differences at Treatment Week 5 between seltorexant and placebo were: −2.9 (80% CI [−4.4, −1.5]; *p* = 0.0049) for 20 mg and −0.9 (80% CI [−2.3, 0.6]; *p* = 0.2271) for 40 mg (Fig. 2A).

#### Changes in HDRS_17_ sleep adjusted total score and HAM-D_6_ subscale score over treatment period: eITT analysis set

Changes in HDRS_17_ sleep adjusted total score and HAM-D_6_ subscale score (analyzed with the same MMRM model used for HDRS_17_ total score) showed LS mean differences at week 5 between the seltorexant 20 mg and placebo arms that trended similarly (nominal p = 0.0289 and 0.0853, respectively) as those obtained for the full HDRS_17_ scores (Fig. 2A). The LS mean differences at week 5 in the seltorexant 20 mg arm was numerically larger than those in the 40 mg arm for both efficacy variables (Fig. 2A).

#### Changes in HDRS_17_ total score at treatment week 5 by subgroups with hyperarousal

In the subgroups of patients with subjective sleep disorder at screening (n = 50), ISI score > 15 at Lead-in Baseline (n = 59), or RRS score≥50 at treatment baseline (n = 50), larger LS Mean differences versus placebo were observed compared to the subgroups of patients from the eITT population who manifested, respectively, the absence of sleep disorder, ISI score ≤ 15, or RRS score < 50 (Fig. 2B). In each of the subgroups manifesting hyperarousal, the LSMean difference relative to placebo appeared numerically larger for the seltorexant 20 mg arm than for the 40 mg arm (Fig. 2B). Notably, the largest LSMean difference versus placebo was observed in patients with RRS ≥ 50 treated with 20 mg: −5.06 with 80%CI [−7.04, −3.07].

#### Clinical response defined by 50% improvement in HDRS_17_ total score

The percentages of patients in the eITT population who manifested an antidepressant response, defined conventionally by ≥50% improvement in the HDRS_17_ total score at Treatment Week 5, were 30.8% for the 20 mg, 29.2% for the 40 mg, and 10.3% for the placebo groups (Supplemental Fig. 1).

#### Remission rates defined by achieving HDRS_17_ total score ≤ 7

At Treatment Week 5 the percentages of patients in the eITT population who achieved remission, defined conventionally by HDRS_17_ total score ≤ 7, were 26.9% for the 20 mg, 29.2% for the 40 mg, and 13.8% for the placebo groups (Supplemental Table 1).

#### Changes in self-rated QIDS-SR16 over treatment period

In the eITT population, the mean (SD) change in QIDS-SR16 total score from baseline to Treatment Week 5 was −3.7 (4.84) for the 20 mg group, −3.0 (4.77) for the 40 mg group, and −1.7 (4.44) for the placebo group.

#### Changes in RRS over treatment period

The mean (SD) RRS total score at treatment baseline was 52.5 (12.05) for placebo group, 54.7 (14.51) for seltorexant 20 mg group, and 53.9 (11.37) for seltorexant 40 mg group (Supplemental Table 2). The mean (SD) reduction in total RRS scores from Treatment baseline to treatment Week 5 was numerically greater in the seltorexant groups: −6.7 (12.31) for seltorexant 20 mg and −6.7 (9.45) for seltorexant 40 mg, compared to the placebo group at −3.8 (7.35) (Supplemental Table 3).

#### Cortisol waking response

**Fig. 3:**
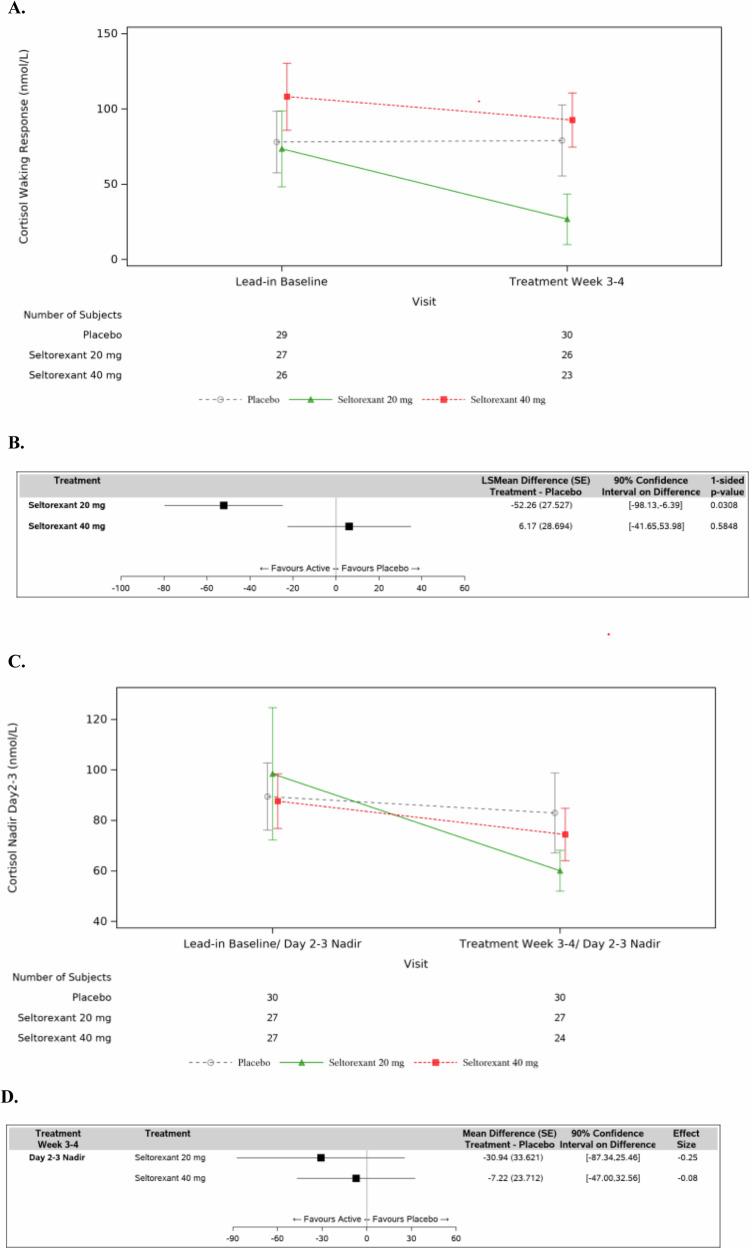
Cortisol waking response and Nadir levels. **A** Mean Cortisol Waking Response (±SE); eITT Analysis Set. **B** Cortisol Waking Response (±SE); Least Squares Mean Changes (in nmol/L) and Comparison Versus Placebo; eITT Analysis Set. **C** Mean Cortisol Nadir Levels (±SE) Over Time (nmol/L); eITT Analysis Set. **D** Cortisol Nadir Levels: Mean Changes (in nmol/L) From Time-Matched Lead-In Baseline and Comparison Versus Placebo at Week 3–4; eITT Analysis Set.

The cortisol waking response numerically decreased in both active treatment groups, with a larger magnitude of reduction seen in the seltorexant 20 mg group: the mean changes from lead-in baseline (SD) at Treatment Week 3/4 were −52.0 (132.98) nmol/L for seltorexant 20 mg, −15.6 (138.44) nmol/L for seltorexant 40 mg, and 0.9 (122.44) nmol/L for placebo (Fig. 3A). Based on the ANCOVA model the difference from placebo was significant for the seltorexant 20 mg group, but not for the seltorexant 40 mg group, with estimated LS Mean differences at Treatment Week 3/4 between seltorexant relative to placebo being −52.3 (90% CI [−98.1, −6.4]; *p* = 0.0308) for 20 mg and 6.2 (90% CI [−41.7, 54.0]; *p* = 0.5848) for 40 mg (Fig. 3B).

#### Cortisol Nadir levels

The mean cortisol nadir levels at Treatment Week 3/4 vs Lead-in baseline numerically trended lower in all three treatment groups (Fig. 3C). The magnitude of reduction was numerically larger in the seltorexant 20 mg group: the mean change from lead-in baseline (SD) at Treatment Week 3/4 was −37.4 (137.80) nmol/L for seltorexant 20 mg, −13.7 (62.39) nmol/L for seltorexant 40 mg, and −6.5 (109.55) nmol/L for placebo (Fig. 3D).

#### PSG assessments

In the eITT population, the mean changes from baseline in the main PSG parameters (Latency to Persistent Sleep [LPS], WASO, TST, and SE) at Treatment Week 3/4 indicated greater improvement in participants treated with 20- or 40-mg seltorexant than in participants treated with placebo (Supplemental Table 4). For both seltorexant doses the reductions in LPS and the increases in TST and SE showed effect sizes >0.5 along with 90% CI which did not overlap 0. For the sleep parameters TST, LPS, WASO, and SE, the mean changes from baseline and mean changes versus placebo were numerically larger in the seltorexant 40 mg group than in the seltorexant 20 mg group.

**Fig. 4:**
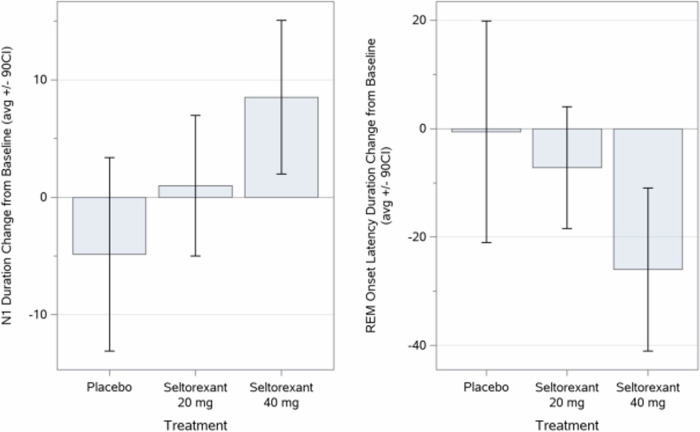
Mean changes in N1 sleep duration and REM latency (in Minutes): eITT analysis set. N1 sleep duration (minutes) and REM onset latency (minutes) duration change from baseline at week 3–4: Mean values (±90% CI).

Notably, patients treated with 40 mg seltorexant spent significantly more time in stage N1 sleep post-treatment, suggesting an increase in time spent in this light sleep stage. In the eITT Analysis Set the mean (SD) change from baseline in time spent in Stage N1 at Treatment Week 3/4 was 0.98 (18.27) minutes for seltorexant 20 mg, 8.52 (19.14) minutes for seltorexant 40 mg, and −4.87 (26.56) minutes for placebo (Fig. 4).

Also noteworthy, the time from sleep onset to REM onset (i.e., REM latency) decreased significantly in the 40 mg seltorexant group but did not change significantly in the 20 mg or placebo groups. In the eITT Analysis Set, the mean (SD) change in REM latency from baseline to Treatment Week 3/4 was −7.17 (34.27) min for seltorexant 20 mg, −26.00 (44.01) min for seltorexant 40 mg, and −0.57 (64.72) min for placebo (Fig. 4).

### Safety

#### Adverse events

The incidence of ≥1 treatment-emergent adverse events (TEAE) in the full safety analysis set was 45.2% (19/42) for 20 mg, 61.0% (25/41) for 40 mg, and 47.7% (21/44) for placebo (Table 2). The most common TEAEs during the Treatment phase were headache (7.1% [3/42] in the 20 mg, 14.6% [6/41] in the 40 mg, and 22.7% [10/44] in the placebo group) and nasopharyngitis (9.5% [4/42] in the 20 mg, 12.2% [5/41] in the 40 mg, and 6.8% [3/44] in the placebo group).

**Table 2:** Summary of treatment-emergent adverse events.

	Placebo (N = 44)	Seltorexant (20 mg) (N = 42)	Seltorexant (40 mg) (N = 41)
Total patients with adverse events (%)	21 (47.7)	19 (45.2)	25 (61.0)
Headache (%)	10 (22.7)	3 (7.1)	6 (14.6)
Nasopharyngitis (%)	3 (6.8)	4 (9.5)	5 (12.2)
Abnormal dreams (%)	2 (4.5)	2 (4.8)	4 (9.8)
Sleep paralysis (%)^a^	0	3 (7.1)	3 (7.3)
Fatigue (%)	2 (4.5)	1 (2.4)	4 (9.8)
Nightmare (%)^a^	0	0	4 (9.8)
Influenza-like illness (%)	3 (6.8)	0	1 (2.4)

Summary of treatment-emergent adverse events in ≥5% of patients during the treatment period; full safety analysis set. Percentages calculated with the number of patients in each group as denominator. Reported dictionary version: MedDRA 21.1.

^a^Adverse events of special interest.

No deaths or serious TEAEs occurred. Treatment-emergent AEs leading to study discontinuation were reported in 2 (4.8%) patients in the 20 mg group during the treatment period (1 patient with elevated hepatic enzymes and 1 patient with elevated hepatic enzymes, blood creatinine increased and rhabdomyolysis). No patient in the placebo or 40 mg groups discontinued due to an AE.

During the treatment period, the incidence of AEs of special interest (AESI) were reported as follows: 7.1% (3/42) in the 20 mg, 24.4% (10/41) in the 40 mg, and 4.5% (2/44) in the placebo group. The most common AESIs during the treatment period were sleep paralysis (7.1% [3/42] in the 20 mg group, 7.3% [3/41] in the 40 mg group), abnormal dreams (4.8% [2/42] in the 20 mg group, 9.8% [4/41] in the 40 mg group, 4.5% [2/44] in the placebo group), and nightmare (9.8% [4/41] in the 40 mg group). There were no significant changes across groups on vital signs (including weight), laboratory measures, or ECG.

#### Suicidal ideation/behavior

No patient reported suicidal behavior during the study. The numbers (percent) of participants with suicidal ideation as assessed using the CSSRS were 7 (16.7%) patients at lead-in baseline and 2 (5.4%) at Treatment Week 5 in the seltorexant 20 mg group, 5 (12.2%) patients at lead-in baseline and 1 (2.7%) at Treatment Week 5 in the seltorexant 40 mg group, and 3 (6.8%) patients at lead-in baseline and 3 (7.1%) at Treatment Week 5 in the placebo group.

## Discussion

Results of this study showed a statistically significant and clinically meaningful antidepressant treatment effect for seltorexant monotherapy 20 mg qhs compared to placebo in the mean change from baseline in HDRS_17_ total score at Treatment week 5 in the eITT population of patients with MDD. In subgroups from the eITT population who manifested subjective sleep disorder, based partly on baseline ISI score > 15, or prominent ruminations, based on baseline RRS score ≥ 50, seltorexant showed greater treatment effects than in the patient subgroups with lower levels of sleep disturbance and ruminations.

These results are consistent with findings from a previous study of individuals with MDD in which seltorexant was administered adjunctively to an antidepressant (SSRI/SNRI) to which the participants had shown inadequate response [6]. In both that study and the current study the treatment effect for 20 mg seltorexant had a moderate effect size and was statistically significant, while the corresponding improvement on the 40 mg dose of seltorexant did not reach statistical significance. In both studies the antidepressant effects of seltorexant were more pronounced in patients with moderate to severe insomnia symptoms. Self-assessment of depressive symptoms (QIDS-SR16) reflected subjective improvements in both seltorexant groups compared to placebo that were numerically most prominent in the 20 mg group. Nevertheless, it is noteworthy that in both the current and previous [6] studies the response and remission rates for the 20- and 40-mg seltorexant doses appeared similar, which in both cases were greater than those achieved on placebo.

For sleep as measured by PSG, relative to placebo both the 20- and 40-mg seltorexant doses showed greater reductions in LPS and WASO, along with increases in TST and SE, with the improvements in these parameters being numerically greater for the 40 mg than the 20 mg seltorexant dose. However, participants receiving 40 mg of seltorexant also showed more time spent in the shallower N1 sleep stage and shorter latency to REM sleep, findings not observed in the 20 mg dose group. Considered together with the observation that the 20 mg dose showed a larger antidepressant treatment effect than the 40 mg dose, these data suggest that seltorexant’s antidepressant treatment effect does not depend simply on increasing the total amount of sleep but may additionally involve the restoration and/or maintenance of physiological sleep (see below).

Furthermore, the cortisol measures obtained in this study suggested larger reductions in nadir cortisol values and cortisol waking responses after 3/4 weeks of treatment with seltorexant 20 mg than with 40 mg compared to placebo. The proportionate magnitude of the reduction in cortisol waking response during treatment with seltorexant 20 mg daily versus placebo is comparable to the proportionate difference between individuals with MDD and healthy controls reported by Vreeburg et al. 2009, suggesting that seltorexant 20 mg daily normalized the abnormality in this response seen in MDD [20].

Therefore, the apparent curvilinear dose-response relationship for the antidepressant treatment effect of seltorexant was associated with corresponding differences in biomarker measures [6]. The 20 mg dose, but not the 40 mg dose, significantly reduced the waking cortisol response, presumably correcting an abnormal elevation reported in MDD [20], while the 40 mg dose, but not the 20 mg dose, significantly decreased the REM SOL, putatively moving this parameter in a counterproductive direction in MDD [11]. Potentially related to this latter finding, Feng et al. described an essential role for the orexin-sublaterodorsal tegmental nucleus neural pathway in relieving REM sleep pressure and presented evidence that disruption of this pathway accounts for the disturbance in sleep-onset REM sleep associated with the orexin deficiency state of narcolepsy [23]. These data considered together with the dose-response relationship we identified for the occurrence of reduced SOL suggest the hypothesis that the 40 mg dose may provide excessive OX2R antagonism in some patients, interfering with this pathway’s function in stabilizing REM sleep, while the 20 mg dose appears sufficient to produce antidepressant effects without interfering with the orexinergic role of stabilizing REM sleep. If so, then the larger antidepressant effect at 20 mg qhs conceivably may partly depend on a more optimal restoration and/or maintenance of physiological sleep compared to either 40 mg or 10 mg qhs (the effects of 10 mg qhs in MDD are reported elsewhere [6, 13]).

Seltorexant was well tolerated and showed a good safety and tolerability profile in MDD patients. The incidence of TEAEs in the 20 mg seltorexant treatment arm (45.2%) was comparable to that observed in the placebo arm (47.7%); TEAEs appeared more prevalent in the 40 mg group (61.0%). The most common TEAEs in the seltorexant combined group during the treatment phase were headache (although the incidence of headache was greater in the placebo group) and nasopharyngitis. Sleep paralysis, nightmares, and abnormal dreams were reported as AESIs, and generally were more common in the 40 mg group than the 20 mg group, although these AESIs did not lead to discontinuation of study drug. No death or suicidal behavior was reported in the study.

A meta-analysis of randomized controlled trials involving FDA-approved antidepressant agents [24] reported that compared to placebo, the mean weighted effect-size for these agents was 0.37 (95% CI, 0.33–0.41) in published studies and 0.15 (95% CI, 0.08–0.22) in unpublished studies. In contrast, in the current study, which involved an MDD sample enriched by removing early placebo responders, the effect size for the 20 mg seltorexant dose group was 0.58. Notably, this strong treatment effect was observed despite the design limitation that the 1:1:1 randomization schedule limited the placebo expectancy to only 0.33, a design element which generally is associated with a larger placebo effect in studies of MDD [25, 26].

### Limitations

The study focuses on results in the placebo non-responder population, and although the full ITT (fITT) sample (placebo responder and non-responder population) showed similar trends, the magnitude of the treatment effects was lower in the fITT sample than in the eITT sample. The double-blind placebo lead-in period was used to proactively exclude early placebo responders from the primary efficacy analysis, and to reduce potential effects of expectation and incentive biases from the study [27]. This approach is hypothesized to enhance the likelihood of obtaining a reliable estimate of treatment effect in the enriched population (i.e., based on showing a minimal response to placebo in the blinded lead in phase).

However, disadvantages to this study design include the requirements for a longer study duration and larger sample size compared to a simple parallel design of same statistical characteristics. The longer duration prolongs the time during which depressed patients do not receive active antidepressant treatment while participating in the clinical trial. The requirement for a larger sample size was partly offset by using one-sided statistical tests to improve power by focusing on one tail of the distribution (i.e., in Phase 1b or 2a trials, only improvement relative to placebo is of interest for progressing compounds further in clinical development).

The generalizability of the results was limited by the inclusion of a largely Caucasian participant sample and the initial exclusion of WOCBP, which led to a higher proportion of men than is representative of the general MDD population. An initial preclinical reproductive toxicology study in rats indicated that treatment with seltorexant (25, 150, and 400 mg/kg) was associated with abnormal estrus cycles in individual females and non-dose responsive changes in reproductive performance (lower fertility and lower conception indices). Once these data became available, a safety measure was implemented to preclude enrollment of WOCBP in ongoing seltorexant studies until the results of a second preclinical reproductive toxicology study were obtained to establish the safety margin and characterize the reversibility of the effects observed on fertility and conception indices. In subsequent clinical studies, female participants with MDD, including WOCBP, have received seltorexant in phase 2 and phase 3 studies to assess the generalizability of the observed safety/efficacy data to WOCBP.

## Supplementary information

Supplemental Materials

## Data Availability

The data sharing policy of the Janssen Pharmaceutical Companies of Johnson & Johnson is available at https://www.janssen.com/clinical–trials/transparency. As noted on this site, requests for access to the clinical study data can be submitted through the Yale Open Data Access (YODA) Project site at http://yoda.yale.edu.

## References

[1] Li J, Hu Z, de Lecea L. The hypocretins/orexins: integrators of multiple physiological functions. Br J Pharmacol. 2014;171:332–50. 24102345 10.1111/bph.12415 PMC3904255

[2] Marcus JN, Aschkenasi CJ, Lee CE, Chemelli RM, Saper CB, Yanagisawa M, et al. Differential expression of orexin receptors 1 and 2 in the rat brain. J Comp Neurol. 2001;435:6–25. 11370008 10.1002/cne.1190

[3] Yun S, Wennerholm M, Shelton JE, Bonaventure P, Letavic MA, Shireman BT, et al. Selective Inhibition of Orexin-2 receptors prevents stress-induced ACTH release in mice. Front Behav Neurosci. 2017;11:83. 28533747 10.3389/fnbeh.2017.00083 PMC5420581

[4] Carter ME, Brill J, Bonnavion P, Huguenard JR, Huerta R, de Lecea L. Mechanism for Hypocretin-mediated sleep-to-wake transitions. Proc Natl Acad Sci USA. 2012;109:E2635–2644. 22955882 10.1073/pnas.1202526109 PMC3465396

[5] Price JL, Drevets WC. Neural circuits underlying the pathophysiology of mood disorders. Trends Cogn Sci. 2012;16:61–71. 22197477 10.1016/j.tics.2011.12.011

[6] Savitz A, Wajs E, Zhang Y, Xu H, Etropolski M, Thase ME, et al. Efficacy and safety of Seltorexant as adjunctive therapy in major depressive disorder: a phase 2b, randomized, placebo-controlled, adaptive dose-finding study. Int J Neuropsychopharmacol. 2021;24:965–76. 34324636 10.1093/ijnp/pyab050 PMC8653874

[7] Lamers F, Vogelzangs N, Merikangas KR, de Jonge P, Beekman AT, Penninx BW. Evidence for a differential role of HPA-axis function, inflammation and metabolic syndrome in melancholic versus atypical depression. Mol Psychiatry. 2013;18:692–9. 23089630 10.1038/mp.2012.144

[8] Roth T. Insomnia: definition, prevalence, etiology, and consequences. J Clin Sleep Med. 2007;3:S7–10. 17824495 PMC1978319

[9] Yehuda R, Seckl J. Minireview: stress-related psychiatric disorders with low cortisol levels: a metabolic hypothesis. Endocrinology. 2011;152:4496–503. 21971152 10.1210/en.2011-1218

[10] Young EA, Haskett RF, Grunhaus L, Pande A, Weinberg VM, Watson SJ, et al. Increased evening activation of the hypothalamic-pituitary-adrenal axis in depressed patients. Arch Gen Psychiatry. 1994;51:701–7. 8080346 10.1001/archpsyc.1994.03950090033005

[11] Thase ME. Depression and sleep: pathophysiology and treatment. Dialogues Clin Neurosci. 2006;8:217–26. 16889107 10.31887/DCNS.2006.8.2/mthase PMC3181772

[12] Riemann D. Voderholzer U. Primary insomnia: a risk factor to develop depression? J Affect Disord. 2003;76:255–9. 12943956 10.1016/s0165-0327(02)00072-1

[13] Brooks S, Jacobs GE, de Boer P, Kent JM, Van Nueten L, van Amerongen G, et al. The selective orexin-2 receptor antagonist seltorexant improves sleep: an exploratory double-blind, placebo controlled, crossover study in antidepressant-treated major depressive disorder patients with persistent insomnia. J Psychopharmacol. 2019;33:202–9. 30644312 10.1177/0269881118822258

[14] Lecrubier Y, Bech P. The Ham D(6) is more homogenous and as sensitive as the Ham D(17). Eur Psychiatry. 2007;22:252–5. 17344030 10.1016/j.eurpsy.2007.01.1218

[15] Recourt K, de Boer P, Zuiker R, Luthringer R, Kent J, van der Ark P, et al. The selective orexin-2 antagonist seltorexant (JNJ-42847922/MIN-202) shows antidepressant and sleep-promoting effects in patients with major depressive disorder. Transl Psychiatry. 2019;9:216. 31481683 10.1038/s41398-019-0553-z PMC6722075

[16] Nolen-Hoeksema S. The role of rumination in depressive disorders and mixed anxiety/depressive symptoms. J Abnorm Psychol. 2000;109:504–11. 11016119

[17] Nolen-Hoeksema S, Davis CG. Thanks for sharing that”: ruminators and their social support networks. J Pers Soc Psychol. 1999;77:801–14. 10531672 10.1037//0022-3514.77.4.801

[18] Nolen-Hoeksema S, Parker LE, Larson J. Ruminative coping with depressed mood following loss. J Pers Soc Psychol. 1994;67:92–104. 8046585 10.1037//0022-3514.67.1.92

[19] Rush AJ, Trivedi MH, Ibrahim HM, Carmody TJ, Arnow B, Klein DN, et al. The 16-Item Quick Inventory of Depressive Symptomatology (QIDS), clinician rating (QIDS-C), and self-report (QIDS-SR): a psychometric evaluation in patients with chronic major depression. Biol Psychiatry. 2003;54:573–83. 12946886 10.1016/s0006-3223(02)01866-8

[20] Vreeburg SA, Hoogendijk WJ, van Pelt J, Derijk RH, Verhagen JC, van Dyck R, et al. Major depressive disorder and hypothalamic-pituitary-adrenal axis activity: results from a large cohort study. Arch Gen Psychiatry. 2009;66:617–26. 19487626 10.1001/archgenpsychiatry.2009.50

[21] Bonaventure P, Shelton J, Yun S, Nepomuceno D, Sutton S, Aluisio L, et al. Characterization of JNJ-42847922, a Selective Orexin-2 receptor antagonist, as a clinical candidate for the treatment of insomnia. J Pharmacol Exp Ther. 2015;354:471–82. 26177655 10.1124/jpet.115.225466

[22] Iber C, Ancoli-Israel S, Chesson AL, Quan SF. The AASM manual for the scoring of sleep and associated events: Rules, terminology and technical specifications. Westchester: American Academy of Sleep Medicine; 2007.

[23] Feng H, Qiao QC, Luo QF, Zhou JY, Lei F, Chen Y, et al. Orexin neurons to sublaterodorsal tegmental nucleus pathway prevents sleep onset REM sleep-like behavior by relieving the REM sleep pressure. Research. 2024;7:0355. 38694202 10.34133/research.0355 PMC11062508

[24] Turner EH, Matthews AM, Linardatos E, Tell RA, Rosenthal R. Selective publication of antidepressant trials and its influence on apparent efficacy. N Engl J Med. 2008;358:252–60. 18199864 10.1056/NEJMsa065779

[25] Papakostas GI, Fava M. Does the probability of receiving placebo influence clinical trial outcome? A meta-regression of double-blind, randomized clinical trials in MDD. Eur Neuropsychopharmacol. 2009;19:34–40. 18823760 10.1016/j.euroneuro.2008.08.009

[26] Sinyor M, Levitt AJ, Cheung AH, Schaffer A, Kiss A, Dowlati Y, et al. Does inclusion of a placebo arm influence response to active antidepressant treatment in randomized controlled trials? Results from pooled and meta-analyses. J Clin Psychiatry. 2010;71:270–9. 20122371 10.4088/JCP.08r04516blu

[27] Schmidt ME, Kezic I, Popova V, Melkote R, Van Der Ark P, Pemberton DJ, et al. Efficacy and safety of aticaprant, a kappa receptor antagonist, adjunctive to oral SSRI/SNRI antidepressant in major depressive disorder: results of a phase 2 randomized, double-blind, placebo-controlled study. Neuropsychopharmacology. 2024;49:1437–47. 38649428 10.1038/s41386-024-01862-x PMC11251157

