# Supplementary material for: Treatment effect and safety of seltorexant as monotherapy for patients with major depressive disorder: a randomized, placebo-controlled clinical trial
Source: Mol Psychiatry. 2024 Dec 11;30(6):2427–35. doi: 10.1038/s41380-024-02846-5 (PMC12092288; doi:10.1038/s41380-024-02846-5)
Supplement: Supplementary file 1 — Supplemental Materials [file 41380_2024_2846_MOESM1_ESM.docx]

**Supplemental Table and Figure Legends:**

**Supplemental Table 1:** Remission Rates at Treatment Week 5 (based on post treatment HDRS_17_ of ≤7) eITT Analysis Set

eITT = enriched intent-to-treat; HDRS_17_ = Hamilton Depression Rating Scale 17. *Total number of patients in the eITT analysis set with evaluable assessments.

**Supplemental Table 2:** RRS Total Score at Baseline; eITT Analysis Set

**Supplemental Table 3:** RRS Mean Changes from Baseline; eITT Analysis Set

**Supplemental Table 4**: Polysomnography: Mean Changes from Lead-in Baseline and Comparison versus Placebo at Treatment Week 3-4 by Treatment; eITT Analysis Set

**Supplemental Figure 1:** Proportion of Participants Manifesting a Clinical Response as Assessed by 50% Improvement in HDRS_17_ Total Score; eITT Analysis Set

eITT, enriched intent-to-treat; HDRS17, Hamilton Depression Rating Scale 17; JNJ42847922, seltorexant

**Supplemental Table 1:** Remission Rates at Treatment Week 5 (based on post treatment HDRS_17_ of ≤7) eITT Analysis Set

|  | **Placebo**  **(N = 30)** | **Seltorexant, 20mg**  **(N = 28)** | **Seltorexant, 40 mg**  **(N = 28)** |
| --- | --- | --- | --- |
| **N*** | 29 | 26 | 24 |
| **No remission (%)** | 25 (86.2) | 19 (73.1) | 17 (70.8) |
| **Remission (%)** | 4 (13.8) | 7 (26.9) | 7 (29.2) |

eITT = enriched intent-to-treat; HDRS_17_ = Hamilton Depression Rating Scale 17. *Total number of patients in the eITT analysis set with evaluable assessments.

**Supplemental Table 2:** RRS Total Score at Baseline; eITT Analysis Set

|  | Placebo (N=30) | Seltorexant 20 mg (N=28) | Seltorexant 40 mg (N=28) | Total (N=86) |
| --- | --- | --- | --- | --- |
|  |  |  |  |  |
| **RRS Total Score** |  |  |  |  |
| N | 30 | 27 | 28 | 85 |
| Mean (SD) | 52.5 (12.05) | 54.7 (14.51) | 53.9 (11.37) | 53.7 (12.56) |
| Median | 51.0 | 52.0 | 54.0 | 52.0 |
| Range | (23; 87) | (30; 81) | (34; 79) | (23; 87) |
|  |  |  |  |  |
| **Categorized RRS Total Score** |  |  |  |  |
| N | 30 | 27 | 28 | 85 |
| RRS Total Score >=50 | 18 (60.0%) | 15 (55.6%) | 17 (60.7%) | 50 (58.8%) |
| RRS Total Score <50 | 12 (40.0%) | 12 (44.4%) | 11 (39.3%) | 35 (41.2%) |

**Supplemental Table 3:** RRS Mean Changes from Baseline; eITT Analysis Set

|  | | | | | | | | Change from Baseline | | | | | | | |
| --- | --- | --- | --- | --- | --- | --- | --- | --- | --- | --- | --- | --- | --- | --- | --- |
| **Parameter**  Treatment Analysis Visit | N | Mean | SE | SD | Median | Min | Max | N | Base Mean | Mean | SE | SD | Median | Min | Max |
| **RRS Total Score** | | | | | | | | | | | | | | | |
| **Placebo (N=30)** | | | | | | | | | | | | | | | |
| Treatment | | | | | | | | | | | | | | | |
| Baseline | 30 | 52.5 | 2.20 | 12.05 | 51.0 | 23 | 87 |  |  |  |  |  |  |  |  |
| Week 1 | 30 | 51.4 | 2.26 | 12.36 | 51.0 | 23 | 88 | 30 | 52.5 | -1.1 | 0.81 | 4.42 | -0.5 | -12 | 6 |
| Week 4 | 28 | 49.6 | 2.58 | 13.67 | 47.0 | 23 | 88 | 28 | 52.7 | -3.1 | 1.05 | 5.55 | -5.0 | -13 | 9 |
| Week 5 | 28 | 48.5 | 2.54 | 13.43 | 46.0 | 22 | 86 | 28 | 52.3 | -3.8 | 1.39 | 7.35 | -4.0 | -21 | 16 |
| End Point | 30 | 48.9 | 2.49 | 13.61 | 46.0 | 22 | 86 | 30 | 52.5 | -3.7 | 1.39 | 7.64 | -4.0 | -21 | 16 |
| **Seltorexant  20 mg (N=28)** | | | | | | | | | | | | | | | |
| Treatment | | | | | | | | | | | | | | | |
| Baseline | 28 | 54.6 | 2.69 | 14.25 | 52.0 | 30 | 81 |  |  |  |  |  |  |  |  |
| Week 1 | 27 | 51.9 | 2.42 | 12.58 | 49.0 | 33 | 74 | 27 | 55.4 | -3.5 | 1.81 | 9.41 | -1.0 | -36 | 10 |
| Week 4 | 21 | 47.7 | 3.06 | 14.01 | 48.0 | 26 | 71 | 21 | 50.4 | -2.7 | 1.89 | 8.67 | 0.0 | -24 | 11 |
| Week 5 | 26 | 47.5 | 2.80 | 14.28 | 46.0 | 23 | 81 | 26 | 54.2 | -6.7 | 2.42 | 12.31 | -2.5 | -35 | 11 |
| End Point | 28 | 47.9 | 2.69 | 14.23 | 46.0 | 23 | 81 | 28 | 54.6 | -6.7 | 2.25 | 11.90 | -3.0 | -35 | 11 |
| **Seltorexant  40 mg (N=28)** | | | | | | | | | | | | | | | |
| Treatment | | | | | | | | | | | | | | | |
| Baseline | 28 | 53.9 | 2.15 | 11.37 | 54.0 | 34 | 79 |  |  |  |  |  |  |  |  |
| Week 1 | 27 | 49.9 | 2.31 | 11.99 | 47.0 | 32 | 76 | 27 | 53.7 | -3.8 | 1.21 | 6.30 | -3.0 | -19 | 10 |
| Week 4 | 22 | 51.7 | 3.01 | 14.13 | 52.5 | 22 | 76 | 22 | 56.2 | -4.5 | 1.78 | 8.36 | -4.5 | -34 | 5 |
| Week 5 | 24 | 47.2 | 2.85 | 13.97 | 44.5 | 22 | 71 | 24 | 53.9 | -6.7 | 1.93 | 9.45 | -5.5 | -34 | 7 |
| End Point | 28 | 48.4 | 2.68 | 14.20 | 47.5 | 22 | 76 | 28 | 53.9 | -5.5 | 1.77 | 9.36 | -4.5 | -34 | 7 |

**Supplemental Table 4**: Polysomnography: Mean Changes from Lead-in Baseline and Comparison versus Placebo at Treatment Week 3-4 by Treatment; eITT Analysis Set

| **Analysis Visit**  PSG Parameter Treatment | N | Mean Change from Baseline (SD) | Mean Change to Placebo (SD pooled) | 90% CI for Mean Change to Placebo | Effect Size |
| --- | --- | --- | --- | --- | --- |
| **Treatment Week 3/4** | | | | | |
| Total Sleep Time (TST) (min) | | | | | |
| Placebo | 30 | -10.43 (86.533) |  |  |  |
| Seltorexant 20 mg | 27 | 24.37 (37.583) | 34.80 (67.941) | [-64.05,-5.55] | 0.51 |
| Seltorexant 40 mg | 25 | 30.84 (59.089) | 41.27 (75.354) | [-74.32,-8.22] | 0.55 |
| Sleep Efficiency (EFF) (%) | | | | | |
| Placebo | 30 | -2.20 (18.021) |  |  |  |
| Seltorexant 20 mg | 27 | 5.09 (7.825) | 7.29 (14.149) | [-13.38,-1.20] | 0.52 |
| Seltorexant 40 mg | 25 | 6.47 (12.271) | 8.67 (15.681) | [-15.55,-1.80] | 0.55 |
| Wake After Sleep Onset (WASO) (min) | | | | | |
| Placebo | 30 | 1.05 (55.821) |  |  |  |
| Seltorexant 20 mg | 27 | -13.30 (32.890) | -14.35 (46.415) | [-5.78,34.47] | -0.31 |
| Seltorexant 40 mg | 25 | -17.84 (52.482) | -18.89 (54.334) | [-5.61,43.39] | -0.35 |
| Latency to Persistent Sleep (LPS) (min) | | | | | |
| Placebo | 30 | 19.65 (81.047) |  |  |  |
| Seltorexant 20 mg | 27 | -12.59 (36.918) | -32.24 (64.092) | [4.63,59.86] | -0.50 |
| Seltorexant 40 mg | 25 | -17.36 (26.816) | -37.01 (62.608) | [10.44,63.58] | -0.59 |
| Number of Awakenings | | | | | |
| Placebo | 30 | -1.23 (9.637) |  |  |  |
| Seltorexant 20 mg | 27 | 1.59 (4.932) | 2.83 (7.776) | [-6.18,0.53] | 0.36 |
| Seltorexant 40 mg | 25 | 2.40 (7.483) | 3.63 (8.728) | [-7.50,0.23] | 0.42 |
| Number of REM Blocks | | | | | |
| Placebo | 30 | -0.53 (1.332) |  |  |  |
| Seltorexant 20 mg | 27 | -0.04 (0.980) | 0.50 (1.179) | [-1.01,0.02] | 0.42 |
| Seltorexant 40 mg | 25 | 0.48 (0.918) | 1.01 (1.163) | [-1.52,-0.50] | 0.87 |
| Sleep Onset to R (REM Latency) (min) | | | | | |
| Placebo | 29 | -0.57 (64.718) |  |  |  |
| Seltorexant 20 mg | 27 | -7.17 (34.268) | -6.60 (52.318) | [-16.45,29.64] | -0.13 |
| Seltorexant 40 mg | 25 | -26.00 (44.010) | -25.43 (56.118) | [0.46,50.40] | -0.45 |
| Stage N1 (min) | | | | | |
| Placebo | 30 | -4.87 (26.556) |  |  |  |
| Seltorexant 20 mg | 27 | 0.98 (18.271) | 5.85 (23.014) | [-15.88,4.18] | 0.25 |
| Seltorexant 40 mg | 25 | 8.52 (19.142) | 13.39 (23.490) | [-23.73,-3.04] | 0.57 |
| Stage N2 (min) | | | | | |
| Placebo | 30 | 0.50 (55.184) |  |  |  |
| Seltorexant 20 mg | 27 | 19.20 (33.054) | 18.70 (46.067) | [-38.69,1.28] | 0.41 |
| Seltorexant 40 mg | 25 | 10.72 (34.018) | 10.22 (46.801) | [-30.60,10.16] | 0.22 |
| Stage N3 (Deep Sleep) (min) | | | | | |
| Placebo | 30 | -4.22 (35.871) |  |  |  |
| Seltorexant 20 mg | 27 | -2.24 (26.314) | 1.98 (31.714) | [-15.84,11.88] | 0.06 |
| Seltorexant 40 mg | 25 | 1.12 (31.277) | 5.34 (33.868) | [-20.50,9.83] | 0.16 |
| Stage REM (min) | | | | | |
| Placebo | 30 | -1.85 (33.190) |  |  |  |
| Seltorexant 20 mg | 27 | 6.43 (23.637) | 8.28 (29.068) | [-20.96,4.41] | 0.28 |
| Seltorexant 40 mg | 25 | 10.48 (23.932) | 12.33 (29.362) | [-25.26,0.60] | 0.42 |
| Time First Awakening After Sleep Onset (min) | | | | | |
| Placebo | 28 | 25.41 (87.260) |  |  |  |
| Seltorexant 20 mg | 23 | 19.83 (162.198) | -5.58 (126.520) | [-58.14,69.31] | -0.04 |
| Seltorexant 40 mg | 25 | 46.30 (125.287) | 20.89 (106.854) | [-71.34,29.56] | 0.20 |
| Note: Treatment baseline is defined as Lead-in baseline value. | | | | | |

**Supplemental Figure 1:** Proportion of Participants Manifesting a Clinical Response as Assessed by 50% Improvement in HDRS_17_ Total Score; eITT Analysis Set


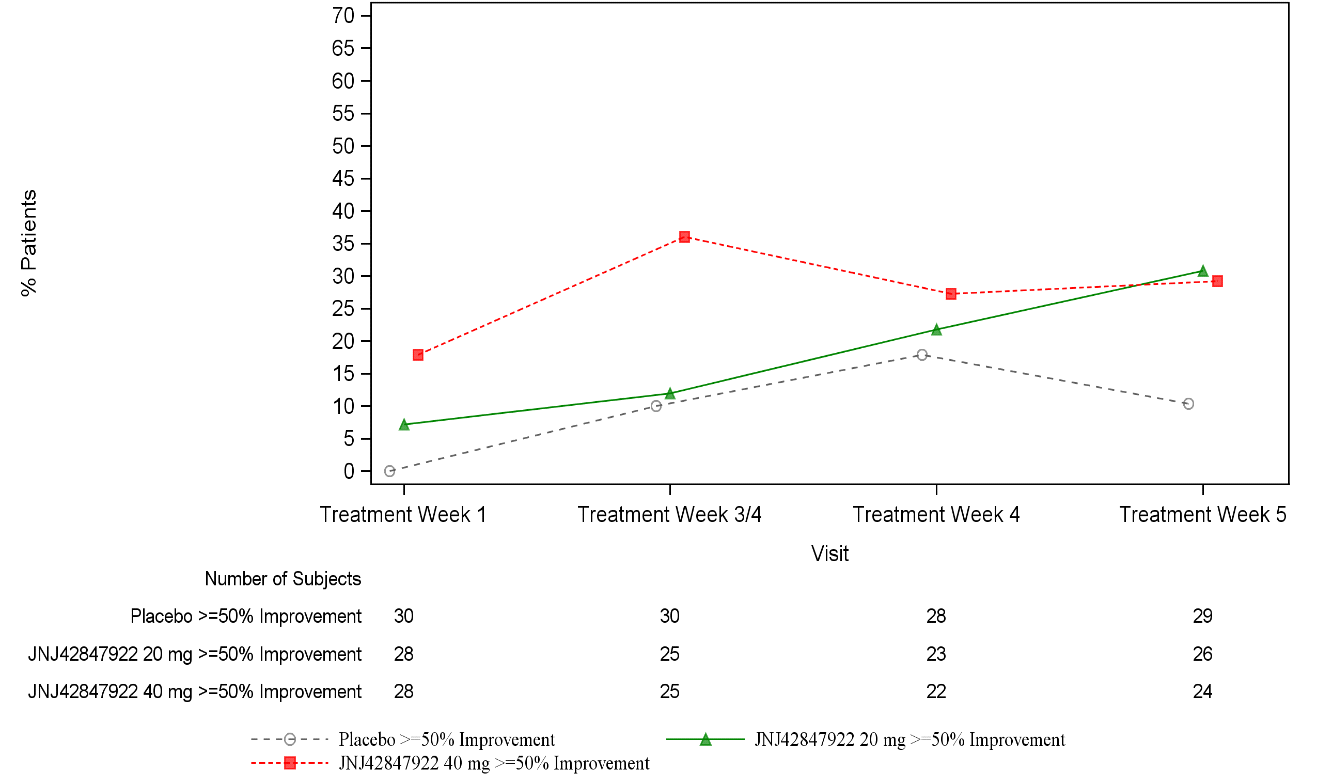


eITT, enriched intent-to-treat; HDRS_17_, Hamilton Depression Rating Scale 17; JNJ42847922, seltorexant
